# Medical Jurisprudence

**Published:** 1850-09

**Authors:** 


*Medical Jurisprudence.* By ALFRED TAYLOR, F. R. S., Licentiate of the Royal College of Physicians, Member of the Royal College of Surgeons, Professor of Medical Jurisprudence and Chemistry in Guy's Hospital. *Second American from the Third London edition, with notes and additions by* R. EGLESFELD GRIFFITH, M. D. Philadelphia. Lea & Blanchard.

The editing of the present American edition was among the last, if not the very last, of the labors of the lamented Griffith, the circumstances of whose death, and a short account of whose labors we presented to our readers in a recent number. We regret that the crowded state of our pages forbids our doing more than simply announcing a new edition, in which a large portion of the whole work has been re-written, and eight new chapters added. Much valuable matter has been introduced in the chapters on Wounds, Infanticide, Pregnancy and Abortion, and the whole may now be considered as one of the best expositors of the existing state of the science both abroad and in our own country.

